# Ataxia and peripheral nerve hypomyelination in ADAM22-deficient mice

**DOI:** 10.1186/1471-2202-6-33

**Published:** 2005-05-06

**Authors:** Koji Sagane, Kazuhiro Hayakawa, Junko Kai, Tomoko Hirohashi, Eiki Takahashi, Norimasa Miyamoto, Mitsuhiro Ino, Tohru Oki, Kazuto Yamazaki, Takeshi Nagasu

**Affiliations:** 1 Tsukuba Research Laboratories, Eisai Co., Ltd., Tokodai 5-1-3, Tsukuba, Ibaraki, 300-2635, Japan; 2 Kawashima Research Laboratories, Eisai Co., Ltd., Kawashimatakehaya-machi 1, Kakamigahara, Gifu, 501-6195, Japan

## Abstract

**Background:**

ADAM22 is a member of the ADAM gene family, but the fact that it is expressed only in the nervous systems makes it unique. ADAM22's sequence similarity to other ADAMs suggests it to be an integrin binder and thus to have a role in cell-cell or cell-matrix interactions. To elucidate the physiological functions of ADAM22, we employed gene targeting to generate ADAM22 knockout mice.

**Results:**

ADAM22-deficient mice were produced in a good accordance with the Mendelian ratio and appeared normal at birth. After one week, severe ataxia was observed, and all homozygotes died before weaning, probably due to convulsions. No major histological abnormalities were detected in the cerebral cortex or cerebellum of the homozygous mutants; however, marked hypomyelination of the peripheral nerves was observed.

**Conclusion:**

The results of our study demonstrate that ADAM22 is closely involved in the correct functioning of the nervous system. Further analysis of ADAM22 will provide clues to understanding the mechanisms of human diseases such as epileptic seizures and peripheral neuropathy.

## Background

ADAM (A Disintegrin And Metalloprotease) is a family of membrane-spanning multi-domain proteins containing a metalloproteinase-like domain and a disintegrin-like domain. Currently, more than 30 ADAMs have been identified in mammals. Their biological activities implicate ADAMs in fertilization, myogenesis and neurogenesis by proteolysis and adhesion. Some types of ADAM are catalytically active metalloproteases and shed the extracellular domains of membrane-bound growth factors or receptors [1,2]. For example, ADAM17 (TACE) has been shown to cleave several substrates, including tumour necrosis factor alpha[3,4], heparin-binding epidermal growth factor-like growth factor [6,7] and transforming growth factor alpha [8]. Studies of ADAM17-null mice have revealed that ADAM17 is critical in embryogenesis and plays an essential role in the supply of growth factors [6,8]. ADAMs are also involved in cell-cell or cell-matrix adhesion through their interaction with integrins or syndecans. More than 10 ADAMs have been shown to support integrin-mediated cell adhesion *in vitro *[9]. It has been reported that ADAM2-null and ADAM3-null male mutants are infertile and their spermatozoa lack egg-binding abilities [10-12]. Both ADAM2 and ADAM3 are not metalloproteases because they lack catalytic site sequences in their metalloprotease domain. These studies clearly showed that non-proteinase members of ADAMs also have significant roles *in vivo*.

We have reported the findings of ADAM11, ADAM22 and ADAM23 genes and their restricted expression in the human and murine nervous systems [13-15]. Sequence analysis suggests that they are not metalloproteases, since they all lack a catalytic motif. It has been reported that ADAM23 protein is localised to the cell surface [16], interacts with alpha-v beta-3 integrin heterodimer [17] and the disruption of ADAM23 gene in the mouse results in premature death associated with ataxia and tremor [18]. Although the cause of death in this mouse is unknown, for these phenotypes, impaired cell-cell or cell-matrix interactions in the nervous system caused by loss of ADAM23 may be responsible. ADAM22 and ADAM23 share highly homologous sequences in their extracellular domains. Especially, it is evident in their putative integrin binding loop sequences, CR(E/D)AVN(E/D)CD, which is located centre of the disintegrin domain. These findings led us to hypothesize that ADAM22 is an integrin binder and plays an important role in the nervous system, as does ADAM23. To determine the physiological functions of ADAM22, we generated and analysed *Adam22 *gene-targeted mice.

## Results

### Generation of ADAM22-deficient mice

**Figure 1:**
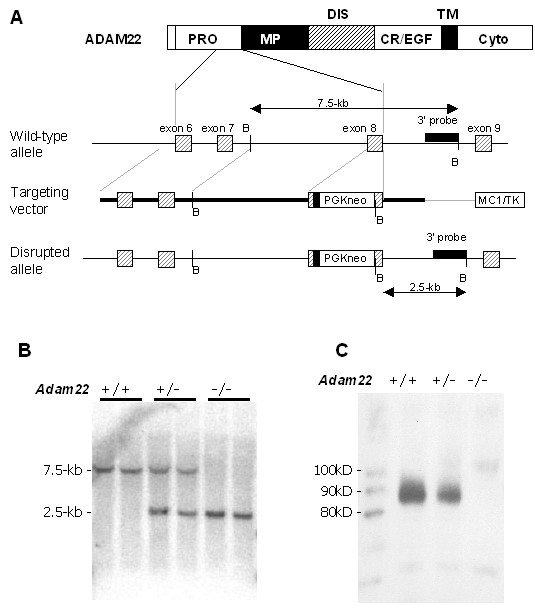
**Targeted mutation of *Adam22*. **(A) The genomic structure of the wild-type *Adam22 *allele (top), the targeting construct (middle) and the disrupted allele (bottom) are shown. ADAM22 expression was disrupted by the insertion of a termination codon and a PGKneo cassette into exon 8. An MC1/TK cassette at the end of the targeting vector allows for negative selection. The 3' probe represents the position of the external probe used for Southern blot analysis, and expected *Bam*HI [B] fragments are indicated by arrows. (B) Southern blot analysis of mouse genomic DNA. The expected DNA fragments for the wild-type allele and disrupted allele are 7.5-kb and 2.5-kb, respectively. +/+, wild-type; +/-, heterozygote; -/-, homozygote. (C) Western blot analysis of ADAM22 expression in the mouse cerebellum. Absence of ADAM22 protein in the *Adam22 *(-/-) mutant cerebellum was shown using anti-ADAM22-cp (cytoplasmic domain) polyclonal antibody.

**Figure 2:**
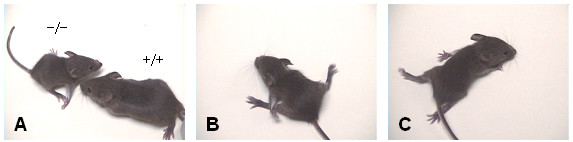
**Uncoordinated movements in the *Adam22 *(-/-) mice at postnatal day 18 (P18). **(A) *Adam22 *(-/-) mice were smaller than their wild-type (+/+) littermates. (B, C) *Adam22 *(-/-) mice at P18 were unable to support themselves on their hindlimbs.

Mice carrying a targeted mutation in their *Adam22 *gene were generated by homologous recombination (Fig. 1A). Correct targeting events were confirmed by Southern blot analysis (Fig. 1B). Since the termination codon was introduced in exon 8 in the pro-protein domain, only the truncated form of the ADAM22 protein would be synthesized from this targeted allele. Because a pro-protein domain is always removed in the mature functional ADAM-proteins by the Furin-like proteases and is thought to be non-functional itself, we considered that this truncated form of ADAM22 protein has no function. Absence of mature ADAM22 protein in homozygous mutants was confirmed by Western blot analysis using the specific antibody, which recognizes the cytoplasmic domain of the ADAM22 protein (Fig. 1C). Homozygous mutants showed no noticeable defects at birth and were indistinguishable from wild-type or heterozygous littermates during the first week. At postnatal day 10 (P10), most of the homozygous mutants were distinguishable by abnormalities such as reduced body weight and uncoordinated movements of their limbs. After P10, all homozygotes displayed severe ataxia (Fig. 2) and began to die. To measure the survival rate and body weight of each genotype precisely, we backcrossed heterozygous male mutants to C57BL/6 females more than 6 times. The resulting (N 6) heterozygous males and females were intercrossed and the produced offspring were analysed. The numbers of survivors of each genotype every 5 days are shown in Table 1. At P10, the ratio of each genotype was in close accordance with the Mendelian ratio (20.5% +/+, 55.1% +/-, 24.4% -/- ; n = 78). This result shows that ADAM22 is not essential for embryogenesis. At P10, the average body weight of homozygous mutants was approximately half that of wild-type and heterozygous littermates (Table 2). Homozygous mutants died one by one after P10, and all homozygotes died before P20. Of more than 100 homozygotes we have produced, none have survived more than 25 days after birth. Meanwhile, heterozygous mutants looked normal, were fertile, and survived for more than one year without obvious defects.

### Histopathology of ADAM22-deficient mice

**Figure 3:**
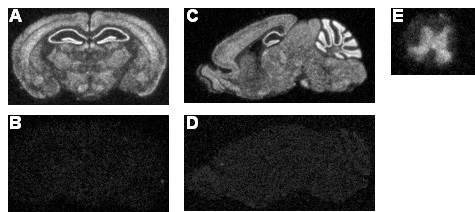
**Neuronal ADAM22 mRNA expression in the CNS. **To determine the ADAM22 mRNA distribution, *in situ *hybridisation analysis using ^35^S-labeled probe was performed. Coronal (A, B) and sagittal (C, D) sections of the mouse brain and spinal cord (E) were shown. Using the antisense probe (A, C), strong signals were obtained, especially in the hippocampus and the cerebellum, while no signals was detected by the sense probe (B, D). In the spinal cord, autoradiograms of ADAM22 mRNA was detected in the grey matter (E).

We have reported the predominant expression of ADAM22 mRNA in the human and mouse brain by Northern blot analysis [13,14]. To determine the distribution of ADAM22 transcript in the adult mouse CNS precisely, *in situ *hybridisation analysis was performed using 35S-labeled RNA probe. As shown in Fig. 3, ADAM22 mRNA was expressed throughout the adult mouse CNS. Strong signal was detected in the cerebellar granule cells and hippocampal formation. In the spinal cord, hybridisation signal was restricted to the grey matter. The distribution of ADAM22 transcripts in the CNS was quite similar with that of ADAM11, whose neuronal expression has been reported [19]. These results suggest that the ADAM22 mRNA expression is neuronal in the mouse CNS.

**Figure 4:**
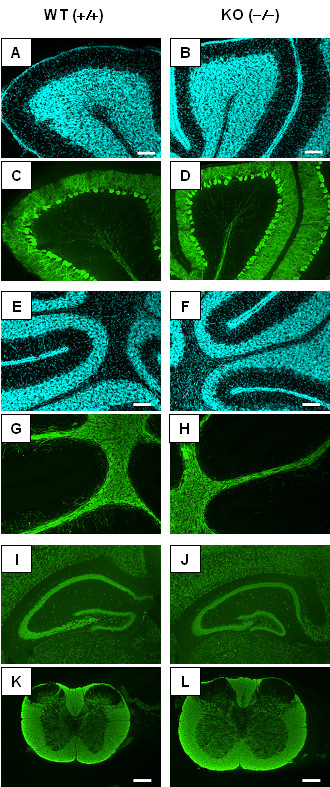
**Normal neurodevelopment in the CNS of ADAM22-deficient mice. **Sagittal sections of the cerebellum from wild-type mice (A, C, E, G) and homozygous mutants (B, D, F, H) at postnatal day 13 were stained for calbindin (C and D; green) or MBP (G and H; green), and were counterstained with DAPI (A, B, E, F; blue). Significant abnormalities were not observed in the homozygotes. Hippocampal neurons were stained by anti-Neu N antibody (I and J). Spinal myelin was analysed by MBP staining (K and L). These analyses showed no obvious differences between homozygotes (J, L) and wild-type littermates (I, K). Bar: (A, B, E, F) 100 μm, (K, L) 250 μm.

Based on the mRNA distribution pattern, we performed immunohistopathological analysis of the cerebellum and hippocampus extensively. Despite the high level of expression of ADAM22 mRNAs in the cerebellar granule cells, granule cell layer was normally formed and Purkinje cell morphology (calbindin-staining) of the mutant mouse looked intact (Figs. 4A–D). Hippocampal formation was also normally formed (Figs. 4I–J). These results suggest that neuronal cell migration was not impaired in the mutant mouse. Myelin-formation detected by MBP (myelin basic protein) staining of the mutant in the cerebellum (Fig. 4H) and spinal cord (Fig. 4L) was also indistinguishable with the wild-type littermate (Figs. 4G,K). In summary, we could not find any signs of abnormalities in the mutant mouse brain by the light microscopic examination.

**Figure 5:**
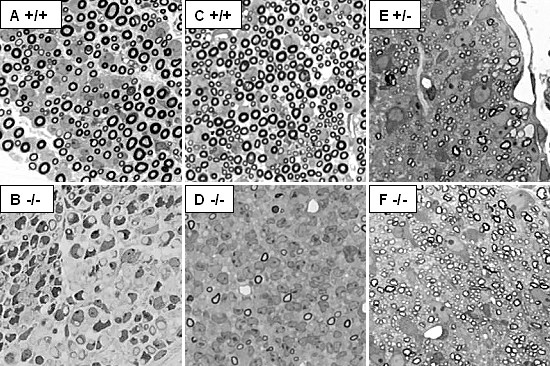
**Hypomyelination of peripheral nerves in ADAM22-deficient mice. **Epon embedded semithin cross-sections of the sciatic nerves (A, B), the trigeminal nerves (C, D) and the spinal cord [lateral funiculus] (E, F) of the indicated genotypes at postnatal day 10 were stained with toluidine blue. Note that the ADAM22-deficient mouse shows thin myelin or lack of myelin in the peripheral nerve fibres (B, D), but the spinal myelinated fibres are intact (F).

**Figure 6:**
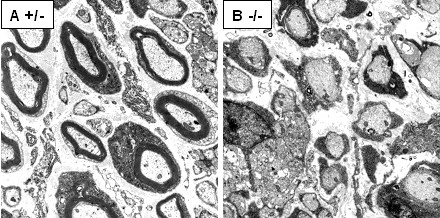
**Electron microscopic analysis of sciatic nerves. **Electron micrographs of the sciatic nerves from *Adam22 *+/- (A) and *Adam22 *-/- (B) mice at postnatal day 10 are shown. In the heterozygote (A), thick myelin was formed, while no myelin was formed in the ADAM22-deficient mouse (B). The axons looked normal in each genotype.

**Figure 7:**
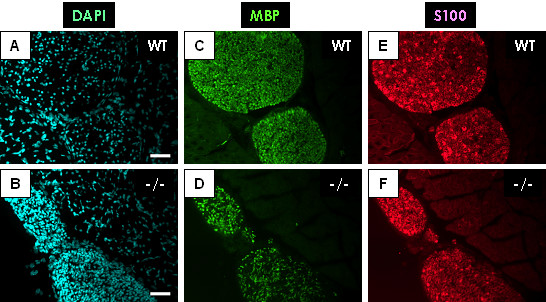
**Marked delay in differentiation of the mutant Schwann cells. **Transverse sections of the sciatic nerves of the indicated genotypes at postnatal day 13 were stained for MBP (C and D; green) or S100 (E and F; red), and were counterstained with DAPI (A, B; blue). In the mutant sciatic nerve, density of DAPI-signals were greatly increased (B) compared with those of the wild-type (A). Bar: (A, B) = 50 μm.

Next, the spinal cord and peripheral nerves of each genotype were analysed by toluidine blue stain, which reveals myelin formation. Surprisingly, lack of myelin or thin myelin was observed in the sciatic nerves (Fig. 5B) and trigeminal nerves (Fig. 5D) in homozygous mutants. Because no lesions were observed in the spinal cord (Fig. 5F), it was suggested that Schwann cell specific myelination defect occurred in the homozygous mice. To analyse the state of myelinating Schwann cells and axons, electron microscopic (EM) analysis of the sciatic nerve was performed (Fig. 6). Schwann cells formed thin or no myelin in the homozygous mutant (Fig. 6B). In contrast, heterozygous mice showed complete myelination (Fig. 6A). Morphology of the axons looked normal in each genotype. Immunohistochemical analysis of the sciatic nerve showed that increased number of nuclei (Fig. 7B) and reduced staining of MBP (Fig. 7D) were remarkable in the homozygous mutant. The Schwann cell marker, S100 staining signal was intensely observed in the homozygote as well as wild-type (Figs. 7E, F). These results suggest that proliferation of the Schwann cells was not impaired but differentiation is severely delayed in the ADAM22-deficient mice.

### Novel ADAM22 transcript variants isolated from the peripheral nervous system

**Figure 8:**
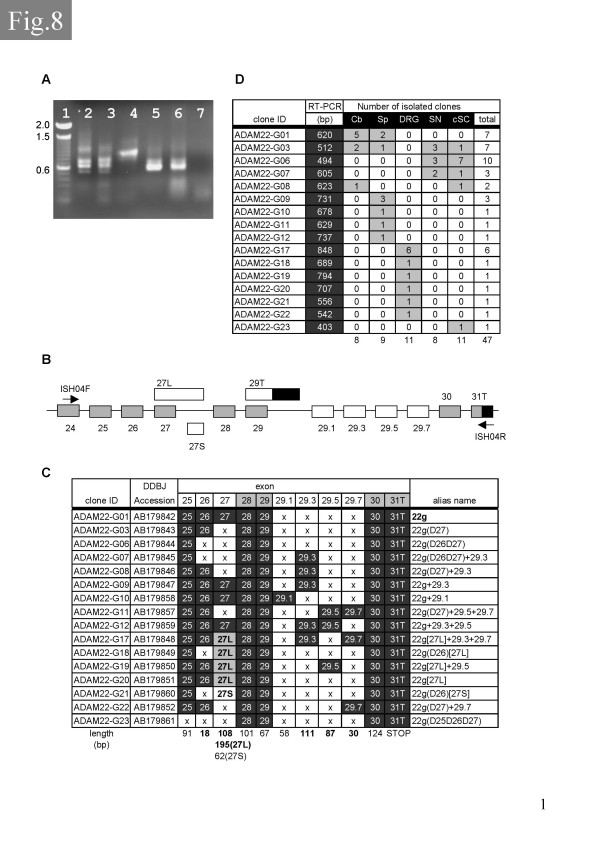
***Adam22 *gene structure and tissue specific transcripts. **(A) RT-PCR analysis. Amplified DNA fragments were analysed by 1 % agarose gel electrophoresis. Lanes 1. 100 bp DNA ladder; 2. cerebellum; 3. spinal cord; 4. dorsal root ganglion; 5. sciatic nerve; 6. cultured Schwann cells; 7. distilled water (B) Exon organization of the mouse *Adam22 *gene is illustrated. Boxes indicate exons. The G01 transcript (orthologous to the human ADAM22 isoform 1 transcript) is composed of grey boxes. Boxes in black indicate non-coding region. (C) Summary of the isolated clones. The nucleotide sequence data have been deposited with the DDBJ/EMBL/GenBank Data Libraries under the accession numbers described in the table. (D) Number of clones isolated from each tissue is summarized. Cb; cerebellum, Sp; spinal cord, DRG; dorsal root ganglion, SN; sciatic nerve, cSC; cultured Schwann cells.

In the case of human ADAM22, isolation of five splicing variants and existence of two terminating exons have been reported [13,20,21]. However, the latter terminating exon (exon 31) of the mouse species has not been identified yet. To isolate the long form of mouse ADAM22 transcripts, at first, we sought the mouse genome by BLAST homology search. Search results showed that the mouse genome contig NT_039297 contained most of the mouse *Adam22 *gene. In this contig sequence, we found putative exons 30 and 31, those sequences were quite similar to human ADAM22 isoform 1 (GenBank # NM_021723). To confirm existence of transcripts, we designed primers on the putative exon 31 and performed RT-PCR using mouse cerebellum mRNA as a template. TA-cloning of the amplified fragments and sequencing analysis showed that 5 of 8 clones were type 1 isoform (G01), 2 clones were type 3 isoform (G03) and 1 clone (G08) contained a novel exon between exons 29 and 30. Next, we performed RT-PCR analysis of mRNAs purified from several adult mouse tissues, such as the spinal cord, dorsal root ganglion (DRG), sciatic nerve and cultured primary Schwann cells. Interestingly, multiple DNA fragments were amplified from each tissue (Fig. 8), and length of major fragments in each lane was different. For example, length of major amplified PCR fragments from the sciatic nerve (Fig. 8A, lane 5) and cultured Schwann cells (lane 6) were almost identical, and were shorter than those from other tissues. In contrast, major fragments from the DRG (lane 4) were longer than others. We performed TA-cloning and sequencing to analyse exon organization of the amplified transcripts (Fig. 8C). Forty seven clones were isolated in total and were classified in 16 independent clones (G01 – G23). Comparison with the genomic sequence revealed the existence of 8 novel exons (exons 27S, 27L, 29.1, 29.3, 29.5, 29.7, 30 and 31). These novel exons were flanked by well-defined introns that obey the GT-AG rule. Exon organization of each clone was shown in Fig. 8C. Characteristic feature of ADAM22 transcripts is tissue specific insertion or skipping of exons. Because the number of nucleotides of exons 26, 27, 27L, 29.3, 29.5 and 29.7 is a multiple of 3, insertion or skipping of these exons will not change the downstream reading frame. Known peptide motives were not found in the newly isolated sequences. This RT-PCR analysis is summarized as follows: ADAM22 transcripts were roughly subdivided in 3 groups, CNS-form (containing exon 27), DRG-form (containing exon 27L) and Schwann-cell-form (skipping exon 26 and 27). From these results, we concluded that ADAM22 is expressed in a cell-type specific manner, and plays an essential role in myelinogenesis in the PNS.

## Discussion

In the present study, we examined the function of ADAM22 by generating mice that lacked the *Adam22 *gene. ADAM22-deficient mice exhibited severe ataxia and premature death. In contrast, heterozygous mutants were healthy, fertile and survived more than 1 year without obvious abnormalities. The cause of death in the homozygous mutants is not clear; however, it is likely to be due to multiple seizures. This is because convulsive seizures were occasionally observed in homozygotes, after which the suffered mice appeared exhausted and, in most cases, died on the next day. It is known that cortical dysplasia resulting from aberrant brain development causes seizure syndrome [22]. For example, mice lacking the neuron-specific activator of cyclin 5, *p35*, exhibit seizures and a severe neuronal migration defect [23]. Because ADAM22 protein is expressed on the cell surface and is likely to be involved in cell-cell or cell-matrix interactions, we hypothesized that depletion of ADAM22 would generate aberrant neuronal migration. Histochemical analysis was performed to verify this hypothesis. However, no marked abnormalities were observed in the mutant mouse brain, ruling out a critical role for ADAM22 in neuronal migration. Another possible explanation is that the premature death in first 2 weeks of life is caused by the dysfunction of the autonomic nervous system. Especially, nerves which control the breathing would be very important, because the respiration system undergoes significant maturation in the first 2–3 weeks after birth. For example, the myelin-deficient (MD) rat, which carries a point mutation in the proteolipid protein (PLP) gene, exhibits ataxia, tremor, dysmyelination and dies at approximately postnatal day 21. In MD rat, early death is caused by the dysfunction of the brain stem which is essential for autonomic control of respiration during hypoxia [24]. Further study is needed to verify the cause of death in ADAM22-deficient mice.

Another interesting phenotype observed in ADAM22-deficient mice was hypomyelinogenesis. Histopathological analysis revealed marked hypomyelination in the sciatic and trigeminal nerves. However, myelin formation in the spinal cord and the brain was normal. It was therefore evident that hypomyelinogenesis occurred specifically in the peripheral nerves in the knockout mice, suggesting Schwann cell dysfunction. In the mutant sciatic nerve, the density of pro-myelinating Schwann cells (S100-positive, MBP-negative) was markedly higher than that seen in heterozygotes. Higher pro-myelinating Schwann cell density and marked delay in myelin formation indicate that ADAM22 plays an important role in Schwann cell differentiation, but not in proliferation.

Investigations on why hypomyelinogenesis occurred in ADAM22-deficient mice centre on integrin. This is because several ADAMs have been shown to interact with integrins via disintegrin domains [9], and it has been suggested that alpha-6 beta-1 integrin on the Schwann cell plays an important role in myelin formation [25]. In addition, laminin-2 is a key player in peripheral myelin formation, because laminin-2 deficiency causes dysmyelination in mice and humans, and its receptors, alpha-6 beta-1 integrin and dystroglycan, are also shown to be deeply involved in myelin formation [26,27]. The conditional knockout studies of beta-1 integrin and laminin gamma-1 in Schwann cell suggested that both laminin-2 and integrins are essential for segregation of large bundles of axons in the early stage of myelin formation [25,28]. In contrast, the phenotype seen in the ADAM22-deficient mice was quite different: for example, unsorted bundles of axons were not seen in nerves and roots. We assume that ADAM22 partially modulates integrin-mediated signals or is involved at a later stage of myelin formation.

## Conclusion

In summary, we generated ADAM22-deficient mice and proved that ADAM22 plays an essential role in both the CNS and the PNS. The results of this study suggest that ADAM22 is involved in human diseases such as epilepsy and peripheral neuropathy. The human ADAM22 gene is relatively large, at 300 kb in length, and is comprised of more than 30 exons. It is assigned on 7q21, where several chromosomal aberrations that are accompanied by neurological disorders have been identified [29,30]. Further detailed molecular function analysis of ADAM22 may lead to progress in uncovering the mechanisms that underlie certain human neurological diseases.

## Methods

### Construction of an Adam22 gene-targeting vector

The 18-kb genomic DNA fragments covering exons 5, 6, 7, 8 and 9 of the *Adam22 *gene were amplified from C57BL/6 genomic DNA by PCR using Expand Hi-Fidelity enzyme mix (Roche Diagnostics) and primers designed for each exon. To generate a mutation in the mouse *Adam22 *gene, we inserted the PGKneo cassette into exon 8 of the *Adam22 *gene. The final targeting construct consisted of a 10.5-kb genomic DNA fragment that was interrupted by the insertion of the PGKneo cassette and contained a MC1/TK cassette as a negative selection marker against random integration [5].

### Generation of Adam22 deficient mice

The linearised targeting vector was electroporated into TT2 embryonic stem (ES) cells [31]. Homologous recombinants were selected by PCR. The extracted genomic DNA from each clone was amplified using the forward primer AGN2: 5'-GCCTGCTTGCCGAATATCATGGTGGAAAAT-3' in the PGKneo cassette, and the reverse primer MFP065R: 5'-ACTATTTCTGTGATGAGGGCACAGCATC-3' outside the targeting vector. Homologous recombined DNA was efficiently amplified by 35 cycles of the following amplification steps (94°C-30 s and 68°C-5 min). The targeting efficiency of this construct was about 4%. Correctly targeted ES clones were injected into fertilized ICR mouse eggs at the eight-cell stage. The resulting male chimeras were mated with C57BL/6N females, and heterozygous male and female mice were interbred to generate homozygous mice. The ataxic phenotypes of homozygous mice were observed in two independent lines.

### Southern blot analysis

Mouse genomic DNA used for Southern blot analysis was extracted from the liver of each genotype. *Bam*HI-digested genomic DNA was probed with the [^32^P]-labelled 0.4-kb *Spe*I-*Bam*HI genomic fragment, which is located between exons 8 and 9.

### Antibody production

His-tagged and MBP-fused recombinant protein containing cytoplasmic domain (cp) of human ADAM22 isoform 1 (position: 756–906, 151 amino acids in length) were produced in *E. coli*. His-tagged ADAM22-cp protein was purified in denatured condition using Ni-NTA resin (Qiagen GmbH) and dialysed in PBS. Precipitated protein was recovered and was mixed with Freund's complete adjuvant (Invitrogen), then, the mixture was used for immunization of rabbits. The antiserum raised against the His-tagged ADAM22-cp protein was incubated with MBP-fused ADAM22-cp protein coupled to Affi-Gel 10 beads (Bio-Rad). Beads with bound antibodies were washed in PBS, and the bound antibodies were eluted with 100 mM glycine, pH 3.0, and neutralized immediately. Using the affinity purified antibody, both human and mouse ADAM22 proteins were detected by Western blot analysis. However, unfortunately the antibody was not suitable for immunohistochemical analysis of mouse tissues.

### Western blot analysis

The absence of ADAM22 protein in the *Adam22 *-/- mice was confirmed by Western blot analysis. Briefly, the cerebellum was isolated from a P14.5 mouse of each genotype and homogenized with a Polytron homogenizer in cell lysis buffer (50 mM Tris-HCl, pH 7.5, 100 mM NaCl, 1% NP-40, Complete protease inhibitors [Roche Diagnostics]). After removal of cell debris by centrifugation, the supernatant was separated on 10% SDS-PAGE, and transferred to a nitrocellulose membrane. The blot was then incubated with polyclonal antibody against the cytoplasmic domain of ADAM22 (at 1:1,000). Bound antibodies were visualized with horseradish peroxidase-labelled second antibody and a ECL-plus chemiluminescence detection system (Amersham Biosciences Corp.).

### Primary Schwann cell culture

Primary Schwann cells were prepared from 4-month-old C57BL/6 mice according to Manent's protocol [32] with minor modifications. Briefly, sciatic nerves were removed and incubated for 7 days in the pre-treatment medium, which consisted of D-MEM (high glucose) supplemented with 10% FCS, 50 mg/ml gentamicin (Invitrogen Corp.), 2.5 μg/ml fungizone (Invitrogen Corp.), 2 μM forskolin (EMD Biosciences Inc.) and 10 ng/ml recombinant heregulin-beta1 (R&D Systems). For dissociation, cultured sciatic nerves were cut into pieces and incubated at 37°C for 3 hours in Opti-MEM medium (Invitrogen Corp.) containing 130 U/ml collagenase type I (Invitrogen Corp.) and 0.4 U/ml dispase II (Roche Diagnostics). Dissociated cells were resuspended in the pre-treatment medium and plated on Poly-D-Lysine/Laminin coated plate (BD Biosciences). The purity of the cultured Schwann cells, as determined by indirect immunofluorescence analysis, approached 90 %.

### RT-PCR analysis

Adult C57BL/6 male mice were used in this study. Total RNAs purified from the cerebellum, spinal cord, sciatic nerve, DRG and cultured Schwann cells using TRIzol (Invitrogen Corp.) and RNeasy kit (Qiagen GmbH) were analysed by RT-PCR using SuperScript II and random primer (Invitrogen Corp.), and PCR amplification (40 cycles; 94°C-30 s, 60°C-30 s and 68°C-5 min) with Expand Hi-Fidelity DNA polymerase (Roche Diagnostics) and ADAM22-specific primers. To detect splicing variants in the cytoplasmic domain, a forward primer was placed just upstream of the transmembrane domain and a reverse primer was set on the terminating exon. The primers used in this study were as follows. ISH04-forward: 5'-AACAGGCACTGGACAGGGGCTGAC-3' and ISH04-reverse: 5'-AATGGATGTCTCCCATAGCCTGGC-3'.

### Histopathology

Mice were anesthetized with ethyl ether. Whole-body perfusion by 2% paraformaldehyde-glutaraldehyde solution followed by heparin-included saline was performed. Sciatic nerves, trigeminal nerves, brain and spinal cord were removed and fixed in 10% neutral-buffered formalin. The spinal cord and other nerve blocks were washed and postfixed with 2% osmium tetroxide, and were dehydrated in ethanol and equilibrated in Epon. Epon embedded semithin sections were stained with toluidine blue and were subjected to light microscopic examination. For electron microscopic analysis, thin sections were cut using an ultramicrotome, stained with 1.5% uranylacetate in 50% ethanol and 0.8% lead citrate, and analysed using electron microscope. All procedures were conducted according to the Eisai Animal Care Committee's guidelines.

### Immunohistochemistry

Frozen sections were rinsed in 0.1% Triton X-100/PBS at room temperature for 1 hour, and were blocked in BLOCKACE solution (Dai-nippon). The following antibodies were incubated overnight in 0.1% BLOCKACE at 4°C:

monoclonal mouse anti-calbindin 28 K (Sigma, 1:200); monoclonal mouse anti-MBP (SMI-99, Sternberger Monoclonals Inc., 1:50); monoclonal mouse anti-Neu N (CHEMICON, 1:100); rabbit anti-S100 polyclonal (DAKO, 1:200). Nuclei were counterstained with 1 μg/ml DAPI (Sigma). MBP staining was performed after microwave epitope retrieval in 10 mmol/L citrate buffer (pH 6). Sections were incubated for 1 hour with secondary antibodies: Cy3-labeled donkey anti-rabbit IgG (Jackson Immuno Research, 1:200), FITC-labelled donkey anti-mouse IgG (Jackson Immuno Research, 1:200). Sections were photographed with an Olympus microscope (Olympus IX71) equipped with a high-resolution CCD camera.

### RNA *in situ *hybridisation

*In situ *hybridisation on frozen sections were carried out using ^35^S-labelled RNA probes. Briefly, 610 bp of mouse ADAM22 cDNA (position: 1351–1960 from initiating ATG) was cloned into the pBluescript II SK(+) or the pBluescript II KS(+) vector. Using these plasmids as templates, sense and antisense labelled RNA probes were generated by T7 RNA polymerase and [α^35^S]UTP. Frozen brain and spinal cord from 2 month-old C57BL/6 female mice were used in this analysis. Pretreatment, hybridisation, RNase treatment and washing was carried out following the protocol described in the literature [33]. Dehydrated slides were attached to imaging plates for 48 hours and the autoradiograms were analysed using a Bio-Image Analyser (BAS3000, Fuji Photo Film).

## Authors' contributions

KS is leading this project, and performed cDNA cloning, plasmid construction, antibody production, immunohistochemical study, and wrote the manuscript. KH and JK conducted histopathological experiments. TH analysed mRNA distribution by *in situ *hybridisation. ET, NM, MI, TO and KY contributed to the generation of knockout mice. TN supervised the project. All authors read and approved the manuscript.

## Figures and Tables

**Table 1:** Genotyping of the progeny of *Adam22 *heterozygous intercrosses.

	*Adam22 *genotype			
		
	+/+	+/-	-/-	total
Day 10	16	43	19	78
Day 15	16	43	8	67
Day 20	16	43	0	59

From heterozygous intercrosses, 78 mice were produced. Numbers of mice in each genotype were counted on postnatal day 10, 15 and 20. No homozygotes were survived more than postnatal day 20. +/+, wild-type; +/-, heterozygote; -/-, homozygote.

**Table 2:** Body weight of progeny at postnatal day 10.5

genotype	body weight (g)	no. of pups
+/+	6.08 ± 0.61	9
+/–	5.84 ± 0.81	16
–/–	3.12 ± 0.48	7

+/+, wild-type; +/-, heterozygote; -/-, homozygote.
